# Determinants of use of care provided by complementary and alternative health care practitioners to pregnant women in primary midwifery care: a prospective cohort study

**DOI:** 10.1186/s12884-015-0555-7

**Published:** 2015-06-25

**Authors:** Esther I Feijen-de Jong, Danielle EMC Jansen, Frank Baarveld, Evelien Spelten, François Schellevis, Sijmen A Reijneveld

**Affiliations:** Department of Midwifery Science, AVAG, and the EMGO Institute for Health and Care Research, VU University Medical Center, Amsterdam, The Netherlands; Department of Health Sciences, University Medical Center Groningen, University of Groningen, Groningen, The Netherlands; Department of Sociology, Interuniversity Center for Social Science Theory and Methodology (ICS), University of Groningen, Groningen, The Netherlands; National Association for Specialty Training for General Practice and GP Trainers, Utrecht, The Netherlands; Faculty of Medicine, Nursing, and Health Science, Monash University, Melbourne, Australia; Department of General Practice, Elderly Care Medicine/EMGO Institute for Health and Care Research, VU University Medical Center, Amsterdam, The Netherlands; Netherlands Institute for Health Services Research (NIVEL), Utrecht, The Netherlands; Academy of Midwifery Amsterdam-Groningen, Dirk Huizingastraat 3-5, 9713 GL Groningen, The Netherlands; PO Box 196, 9700 AD Groningen, The Netherlands; PO Box 20072, 3502 LB Utrecht, The Netherlands; PO Box 1568, 3500 BN Utrecht, The Netherlands

**Keywords:** Health care utilization, Midwifery care, Maternal health care, Low-risk pregnancy, Complementary and Alternative Medicine (CAM)

## Abstract

**Background:**

Pregnant women visit complementary/alternative health care practitioners in addition to regular maternal health care practitioners. A wide variation has been reported with regard to rates and determinants of use of complementary/alternative medicine (CAM), which may be due to heterogeneous populations. The aim of this study was to examine the prevalence and determinants of use of CAM practitioners by a homogeneous population of low-risk pregnant women in the Netherlands.

**Methods:**

Data from the population-based DELIVER study was used, concerning 1500 clients from twenty midwifery practices across the Netherlands in 2009 and 2010. CAM use was measured based on patient reports. Potential determinants were derived from Andersen’s behavioural model of health care utilization.

**Results:**

The prevalence of CAM practitioner use by low-risk pregnant women was 9.4 %. Women were more likely to use CAM if they had supplementary health care insurance (OR 3.11; CI 1.41-6.85), rated their health as ‘bad/fair’ (OR 2.63; CI 1.65-4.21), reported a chronic illness or handicap (OR 1.93; CI 1.14-3.27), smoked during pregnancy (OR 1.88; CI 1.06-3.33), or used alcohol during pregnancy (OR 2.30; CI 1.46-3.63).

**Conclusions:**

CAM is relatively frequently used by low-risk pregnant women. Determinants revealed in this study diverge from other studies using heterogeneous populations. Maternal health care practitioners must be aware of CAM use by low-risk pregnant women and incorporate this knowledge into daily practice by actively discussing this subject with pregnant women.

## Background

Pregnant women visit complementary/alternative health care practitioners in addition to regular maternal health care practitioners (e.g., obstetricians, midwives and GPs). Complementary/alternative medicine (CAM)—a group of diverse medical and health care systems, practices and products that are not traditionally considered part of conventional medicine [1]—may be used because pregnant women may be concerned about the potentially harmful effects of conventional medicine on their babies or it can be an expression of dissatisfaction with conventional medicine [2, 3]. A wide variation has been reported with regard to the rate of use of CAM during pregnancy, with ranges from 1 to 87 % found in reviews [4, 5]. The available evidence on CAM use among pregnant women mostly covers women in a variety of settings, such as antenatal clinics, gynaecology wards, outreach clinics, local communities and birth clinics [4, 5], which may explain this large variation in rates. Next to this, these studies mostly concern use of CAM (i.e.,: herbal medicine, flower essence etc.) instead of use of CAM practitioners.

Documented determinants of CAM use by pregnant women include completion of tertiary-level education and the use of CAM prior to becoming pregnant [4]. Adams et al.[5] also found that primiparous women, non-smoking women and women planning a natural birth were more likely to use CAM. Steel et al. reported that women who had either a vocational or university qualification were more likely to consult an acupuncturist [6]. Similar to the evidence on prevalence, studies on the determinants of CAM use also concern heterogeneous populations, consisting of a combination of women with low-risk and high-risk pregnancies.

In the general population, it is known that poorer health status predicts CAM use [7]. Since a low-risk pregnancy population in general consists of women who are not known to have any medical or obstetric risk factors before the onset of labour [8], it can be hypothesized that such a specific low-risk population would use less CAM than high-risk or heterogeneous populations. The Dutch maternity health care system provides a very suitable setting to study a homogeneous low-risk pregnancy population. The system is divided into two echelons. In the first, midwives are the main care practitioners for pregnant women who have low-risk pregnancies (primary midwifery care). Of all pregnant women, 85.4 % start prenatal care in this first echelon [9]. Only when problems arise, are pregnant women referred to gynaecologists/obstetricians for secondary care. However, there is close mutual cooperation between these echelons [10]. Regarding health insurance, basic health insurance is obligatory for all Dutch people; however, reimbursement of the costs for midwifery-led hospital births requires supplementary insurance.

Knowledge about CAM practitioner use by women with low-risk pregnancies is needed since this group of pregnant women constitutes the majority of all pregnant women whereas research about this group is lacking. Next to this, the results of this study will provide insight in the health care needs of and potential risks encountered by low-risk pregnant women, i.e., issues that maternal health care practitioners must take seriously [3]. Furthermore, CAM practitioner use can have an impact on women’s health care decision-making during pregnancy. It could be conceived that CAM practitioner use leads to a reduction of use of regular care. D’Crus et al. [11] reported that people choose to consult CAM practitioners to get counseling for general health issues and for another health perspective. Such health care decisions may be associated with changes in health care services use [6].

Although it is frequently assumed that CAM use ‘will do no harm’ [12], evidence regarding safety and efficacy does not fully confirm this [13]. For example, naturopaths, a type of CAM provider can recommend herbal medicine which, in case reports, has been linked to adverse foetal outcomes [14]. Also, Steel et al. reported that consuming herbal teas is associated with a higher likelihood of medical removal of placenta/blood clots [15]. On the other hand CAM can be valuable for pregnant women, for instance, Viljoen et al. concluded in a systematic review that ginger can be considered an option for women suffering pregnancy-associated nausea [16].

The aim of this study was to examine the prevalence and determinants of use of CAM practitioners by low-risk pregnant women in the Netherlands. We used Andersen’s behavioural model of determinants of health care utilization as a guiding framework to categorize these determinants. This model suggests that the use of health care services depends on predisposing, enabling, need and health behaviour factors [17].

## Methods

### Study design

Data for this analysis was obtained from the DELIVER study (Dutch acronym for ‘data primary care delivery’) conducted by the Department of Midwifery Science of VU University Medical Center, Amsterdam. The DELIVER study is a descriptive study that aimed to provide information about the organization of midwifery care, the accessibility of midwifery care and the quality of primary midwifery care in the Netherlands [18].

### Participants, setting and procedure

In the DELIVER study, a two-stage sampling procedure was used. Firstly, midwifery practices were recruited by using purposive sampling. Three stratification criteria were used: region (north, east, south, west), level of urbanisation (urban or rural area), and practice type (dual or group practice) to ensure that different types of practices in different regions were represented. Subsequently, all clients receiving care in the participating primary midwifery practices at any moment in a 12 month study period in 2009–2010 were eligible to participate if they were able to understand Dutch, English, Turkish or Arabic. The participating practices (20 of the 519 midwifery practices in the Netherlands) comprised 110 midwives and a caseload of 8200 clients per year, with all regions of the Netherlands being represented [18].

Clients participating in the DELIVER study completed up to three questionnaires. The first questionnaire was administered before 34 weeks of gestation, the second between 34 weeks of gestation and birth, and the third in the postpartum period. In addition, information was collected about the care provided by midwives by extracting data from electronic client records of participating clients and from the Netherlands Perinatal Registry. The latter consists of information provided by midwives, GPs and obstetricians. Reporting to this Registry is obligatory. The three data sources were linked using unique, anonymous client and midwifery practice identifiers [18]. The Medical Ethics Committee of VU University Medical Center, Amsterdam approved the study protocol of the DELIVER study. Participants provided written informed consent.

**Fig. 1:**
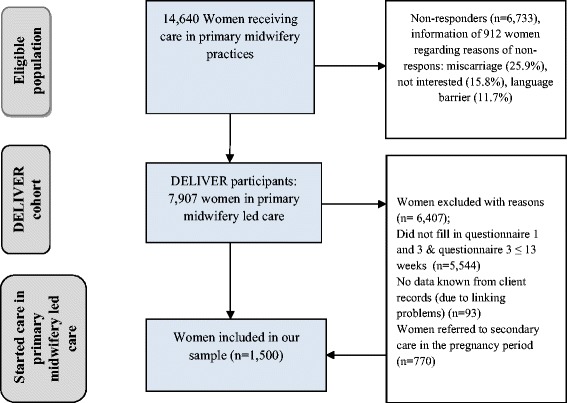
Eligible population, DELIVER cohort and study population

**Fig. 2:**
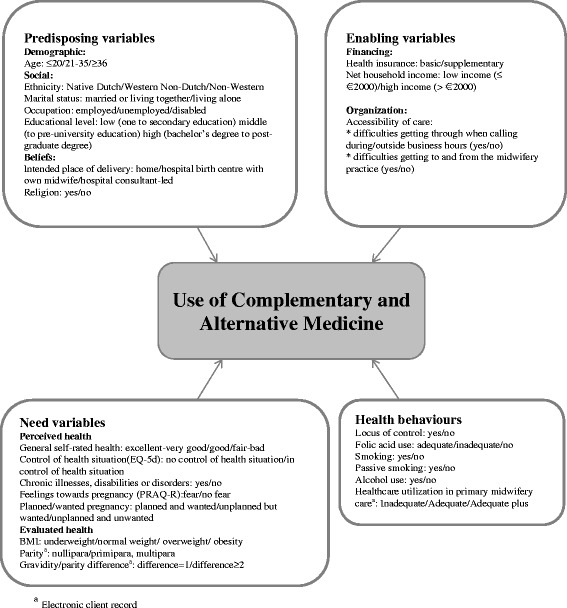
Conceptual framework; Andersen’s behavioural model, which shows the possible determinants of HCU

Our study comprised those pregnant women who filled in the first and third questionnaires (up to 13 weeks postpartum) and whose questionnaire data could be linked to the electronic client record data and the Netherlands Perinatal Registry data. To maximize the homogeneity of the low-risk population, we excluded women who were referred to secondary care during pregnancy. Women who were referred during labour were classified as non-referred because the pregnancies of these women were low-risk (Fig. 1). All women filled in the questionnaires at home without interference from a professional. We used data from electronic client records with regard to two independent variables; 1. Health care utilization in midwifery practices (Fig. 2, health behaviours: ‘health care utilization of pregnant women in primary midwifery care), and 2. Parity (Fig. 2, need variables: ‘parity’). These variables were shown to be invalidly measured in the client questionnaires. We assume that midwives recorded visits to their practice and parities of women more validly.

### Measurements

*CAM practitioner use* was measured by two items in the third questionnaire of the DELIVER study: ‘*Please indicate whether you have seen any of the following practitioners of complementary or alternative medicine since the beginning of your pregnancy’ and* ‘*What other practitioner(s) of complementary or alternative medicine did you see?’* For each practitioner, women had to specify contact rates in predefined categories (0, 1–3, 4–6, 7–9, 10–12, 13–15, >15 meetings). Various types of CAM practitioners were stated explicitly in the questionnaire: acupuncturist, anthroposophical practitioner, homeopath, manual therapist (chiropractor, osteopath, manual therapist), naturopath (diet therapy, neural therapy, herbal therapy) or paranormal practitioner (psychic, faith healer, magnetic therapist), and respondents also had the option to choose other alternative practitioner. Women who reported at least one consultation with a CAM practitioner were defined as CAM users.

Potential determinants of CAM practitioner use concerned *predisposing, enabling, need and health behaviour variables.* Data on possible determinants were obtained from the first questionnaire and the electronic client records. Several variables, based on Andersen’s model [17], were considered to be potential determinants of CAM practitioner use. In the Andersen model use of health services depends on individual and contextual characteristics, and on health behaviour. The following components were measured: predisposing, enabling, need, and health behaviour characteristics. Predisposing characteristics are existing conditions that predispose people to use (yes/no) healthcare services. Enabling/disabling characteristics facilitate or impede use. Need characteristics are conditions that patients or health providers recognize as requiring medical treatment. Health behaviour characteristics are behaviours on the part of the individual that influence health status [17]. Potential determinants were categorized into one of these components by using existing literature of the Andersen’s model and by discussion of the authors.

Operationalizations of the independent variables are shown in Fig. 2.

*Predisposing variables* encompassed socio-demographic and belief factors, consisting of age, ethnicity, marital status, occupation, educational level, intended place of delivery, and religion. *Enabling variables* included finance (health care insurance) and organization (accessibility of care) variables. Regarding health insurance, we distinguished between basic and supplementary health care insurance.

*Need variables* comprised the health status of the client. The descriptive component of EuroQol (EQ) was used to measure self-reported health status [19]. This component asked the respondent to consider and rate her actual health on five dimensions: mobility, self-care, usual activities, pain/discomfort and anxiety/depression. Responses to questions on each of these dimensions can take one of five values, which concern five levels of severity (no problems/slight problems/moderate problems/severe problems/extreme problems). Health status values ranged from extreme problems on all five dimensions (value =-0.109) to no problems on any dimension (value = 1.0). A single health status value was calculated by applying scores from a UK valuation set [19]. We then dichotomized the scores as ‘poor’ (lowest quartile) and ‘the remainder’. Feelings towards pregnancy were measured by using the Pregnancy Related Anxiety Questionnaire (PRAQ) [20]. The scales used were ‘fear of giving birth’ (two items), ‘fear of bearing a handicapped child’ (four items) and ‘concern about one’s appearance’ (three items). Items were scored on a four-point scale (4 = very true, 3 = true, 2 = not true, 1 = certainly not true). Every item score was dichotomized based on the median score. BMI was calculated using the weight and height before pregnancy reported by the respondent. We classified BMI according to the World Health Organization classification of adult underweight, normal weight, overweight and obesity [21]. Finally, we computed a variable ‘gravidity/parity difference’ which measured the difference between the number of pregnancies and the number of deliveries. We hypothesised that there could be a difference in prenatal health care use between women with miscarriage(s) and/or abortion(s) in their obstetric history.

*Health behaviour variables* consisted of questions related to smoking, soft and hard drug use, alcohol use, adequate folic acid use, locus of control and adequacy of prenatal health care utilization of pregnant women in primary midwifery care. We did not include drug use because none of the pregnant women reported drug use, which concurs with our sampling of low-risk pregnancies [18]. The locus of control was measured by a single question about the extent of the perceived possibility of influencing lifestyle and/or health behaviour (‘*To what extent do you feel that you can influence your health by changing your lifestyle and/or behaviour?*’). Folic acid use was labelled as adequate when started at least four weeks before pregnancy [22]. Adequacy of prenatal health care utilization of women in primary midwifery care was measured using the Kotelchuck Index, which is widely used in the US [23]. We constructed a revised assessment index of the adequacy of prenatal care use in Dutch primary midwifery care (Table 1), modified according to the guidelines of the Royal Dutch Organization of Midwives, concerning the number of prenatal visits during pregnancy. This index combines the timing of initial prenatal health care and the number of prenatal health care visits. Prenatal care entry regarded on the gestational age at the first prenatal visit and classified into ‘timely’ (gestational age at onset < 12 weeks) and ‘late’ (gestational age at onset ≥ 12 weeks). The number of prenatal visits was derived from the electronic client record, and compared to the “expected” number of visits as described by the Dutch prenatal guideline for primary midwifery care taking the gestational age at which women gave birth into account. Adequacy of prenatal health care utilization was trichotomized into ‘adequate plus’, ‘adequate’ and ‘inadequate’ (inadequate and intermediate) care.

**Table 1:** Assessment index of the adequacy of prenatal care use in the Dutch primary care context (A.W. Boerleider and E.I. Feijen-de Jong)

Duration of gestation (completed weeks and days of pregnancy, respectively)	Initiation of care	Number of visits	Kotelchuck Index
0-11+6^1^	≤11^+6^	≥3	4
		1–2	3
			2
		0	1
12-26^+6^	≤11^+6^	≥6	4
Ideally 3.75 visits^1^		3–5	3
		2	2
		≤1	1
	≥12^+0^		1
27-36^+6^		≥10	4
Ideally 7.5 visits^1^		6–9	3
		4–5	2
		≤3	1
	≥12^+0^		1
37-37^+6^	≤11^+6^	≥13	4
Ideally 11 visits^1^		10–12	3
		6–9	2
		≤5	1
	≥12^+0^		1
38-38^+6^	≤11^+6^	≥14	4
Ideally 12 visits^1^		10–13	3
		6–9	2
		≤5	1
	≥12^+0^		1
39-39^+6^	≤11^+6^	≥15	4
Ideally 13 visits^1^		11–14	3
		7–10	2
		≤6	1
	≥12^+0^		1
40-40^+6^	≤11^+6^	≥16	4
Ideally 14 visits^1^		12–15	3
		7–11	2
		≤6	1
	≥12^+0^		1
41-41^+6^	≤11^+6^	≥17	4
Ideally 15 visits^1^		12–16	3
		8–11	2
		≤7	1
	≥12^+0^		1

1. Inadequate (received less than 50 % of expected visits)

2. Intermediate (50–79 %)

3. Adequate (80–109 %)

4. Adequate Plus (110 % and more)

^1^According to the guidelines of the Royal Dutch Organization of Midwives

### Statistical analyses

First, we described the background characteristics of the study population, and second the prevalence of CAM practitioner use. Third, we performed univariable logistic regression analyses for all determinants. Next, we performed multivariable logistic regression with a backward selection procedure, i.e., stepwise deletion of the variables that contributed least to the model that predicts use of CAM practitioners until all remaining variables contributed significantly at p < 0.05 level. The results are presented as odds ratios (ORs) and 95 % confidence intervals (CI). Women reporting no use of a CAM practitioner were our reference group. The structure of the data was hierarchical, i.e., respondents were clustered by midwifery practice. Characteristics of practices may affect all women who received care in that practice, which might lead to dependency of data regarding women coming from the same practice [24]. To adjust for this potential clustering, multilevel analytical methods were used. A two-tailed p-value of 0.05 or lower was considered statistically significant. Missing data accounted for less than 1.5 % of all variables, with the exception of 6.5 % for BMI. SPSS 21.0 (SPSS Inc., Chicago, IL) was used for all analyses.

## Results

Our study population included 1500 women with low-risk pregnancies. Table 2 shows the potential determinants and the rate of CAM practitioner use of these women in primary midwifery care. Regarding background variables, the majority of the pregnant women were between 21–35 years of age (85.5 %), native Dutch (88.5 %), married (97.8 %), employed (84.3 %) and higher educated (i.e., having a bachelor’s degree or higher) (55.2 %). Of all the women, 9.4 % reported having consulted a CAM practitioner.

**Table 2:** Use of a complementary/alternative medicine practitioner (CAM) by low-risk pregnant women in primary midwifery care (N = 1500)

	Consultation of a CAM practitioner		
		Yes	No
Background characteristics	N = 1500 (%)	N = 141 (9.4)	N = 1359 (90.6)
Age			
≤20	14 (0.9)	1 (0.7)	13 (1.0)
21–35	1283 (85.6)	118 (83.7)	1165 (85.7)
≥36	202 (13.5)	22 (15.6)	180 (13.3)
Missing	1	0	1
Ethnicity			
Native Dutch	1327 (88.6)	121 (85.8)	1206 (88.9)
Non-Western	65 (4.3)	6 (4.3)	59 (4.3)
Western Non-Dutch	106 (7.1)	14 (9.9)	92 (6.8)
Missing	2	0	2
Marital status			
Married or living together	1467 (97.8)	137 (97.2)	1330 (97.9)
Living alone	33 (2.2)	4 (2.8)	29 (2.1)
Missing	0	0	0
Occupation			
Employed	1264 (84.3)	117 (83.0)	1147 (84.4)
Unemployed	220 (14.7)	22 (15.6)	198 (14.6)
Disabled	16 (1.1)	2 (1.4)	14 (1.0)
Missing	0	0	0
Educational level			
Low	164 (10.9)	11 (7.8)	153 (11.3)
Middle	508 (33.9)	42 (29.8)	466 (34.3)
High	828 (55.2)	88 (62.4)	740 (54.4)
Missing	0	0	0
Intended place of delivery			
Hospital/Birth centre midwifery-led	856 (57.1)	85 (60.3)	771 (56.7)
Hospital consultant-led	19 (1.3)	3 (2.1)	16 (1.2)
Home	625 (41.7)	53 (37.6)	572 (42.1)
Missing	0	0	0
Religion			
No	845 (57.2)	84 (60.0)	761 (57.0)
Yes	631 (42.8)	56 (40.0)	575 (43.0)
Missing	24	1	23
Basic and supplementary health care insurance			
Basic and supplementary	1307 (87.4)	134 (95.0)	1173 (86.6)
Basic	188 (12.6)	7 (5.0)	181 (13.4)
Missing	5	0	5
Net household income^a^			
> €2000	1082 (72.2)	110 (78.0)	972 (71.6)
< €2000	170 (11.3)	11 (7.8)	159 (11.7)
Missing	0	0	0
Consultation of a CAM practitioner		Yes	No
Accessibility of care (phone)			
Problems	252 (16.8)	25 (17.7)	227 (16.7)
No problems	1248 (83.2)	116 (82.3)	1132 (83.3)
Missing	0	0	0
Accessibility of care (getting to and from the practice)			
Problems	65 (4.3)	6 (4.3)	59 (4.3)
No problems	1435 (95.7)	135 (95.7)	1300 (95.7)
Missing	0	0	0
General self-rated health			
Excellent/Very good	538 (35.9)	45 (31.9)	493 (36.3)
Bad/Fair	181 (12.1)	35 (24.8)	146 (10.7)
Good	781 (52.1)	61 (43.3)	720 (53.0)
Missing	0	0	0
Quality of life (EuroQol)			
Poor health status	313 (20.9)	44 (31.2)	269 (19.8)
Good health status	1187 (79.1)	97 (68.8)	1090 (80.2)
Missing	0	0	0
Chronic illnesses or handicaps			
Yes	127 (8.5)	23 (16.3)	104 (7.7)
No	1373 (91.5)	118 (83.7)	1255 (92.3)
Missing	0	0	0
PRAQ-Child			
Fear	508 (33.9)	50 (35.5)	458 (33.8)
No fear	989 (66.1)	91 (64.5)	898 (66.2)
Missing	3	0	3
PRAQ-Delivery			
Fear	19 (1.3)	3 (2.1)	16 (1.2)
No fear	1480 (98.7)	138 (97.9)	1342 (98.8)
Missing	1	0	1
PRAQ-Body			
Fear	418 (27.9)	43 (31.9)	373 (27.5)
No fear	1079 (72.1)	96 (68.1)	983 (72.5)
Missing	3	0	3
Planned and wantedness of pregnancy^b^			
Wanted, not planned	231 (15.4)	24 (17.1)	207 (15.2)
Planned and wanted	1268 (84.6)	117 (83.3)	1151 (84.4)
Missing	1	0	1
BMI			
≤18.5	40 (2.7)	6 (4.3)	34 (2.5)
25- < 30	274 (18.3)	26 (18.4)	248 (18.2)
≥30	72 (4.8)	5 (3.5)	67 (4.9)
18.5- < 25	1017 (67.8)	96 (68.1)	921 (67.8)
Missing	97	8	89
Parity			
Primi/multiparous	653 (43.5)	58 (41.1)	595 (43.8)
Nulliparous	847 (56.5)	83 (58.9)	764 (56.2)
Missing	0	0	0
Difference between number of pregnancies and number of births			
≥2	359 (24.2)	38 (27.1)	321 (23.8)
1	1127 (75.8)	102 (72.9)	1025 (76.2)
Missing	14	1	13
Locus of control			
No	174 (11.6)	13 (9.2)	161 (11.9)
Yes	1325 (88.4)	128 (90.8)	1197 (88.1)
Missing	1	0	1
Folic acid use			
No	110 (7.3)	9 (6.4)	101 (7.4)
Yes, inadequately	683 (45.6)	70 (49.6)	613 (45.2)
Yes, adequately	705 (47.1)	62 (44.0)	643 (47.4)
Missing	2	0	2
Smoking			
Yes	108 (7.2)	17 (12.1)	91 (6.7)
No	1392 (92.8)	124 (87.9)	1268 (93.3)
Missing	0	0	0
Passive smoking			
Yes	173 (11.5)	10 (7.1)	80 (5.9)
No	1326 (88.5)	131 (92.9)	1279 (94.1)
Missing	0	0	0
Alcohol use			
Yes	173 (11.5)	30 (21.4)	143 (10.5)
No	1326 (88.5)	110 (78.6)	1216 (89.5)
Missing	1	1	0
Health care utilization in Midwifery Practice			
Inadequate	384 (25.6)	39 (27.7)	345 (25.4)
Adequate plus	95 (6.3)	6 (4.3)	89 (6.5)
Adequate	1021 (68.1)	96 (68.1)	925 (68.1)
Missing	0	0	0

^a^Missings in a third category (Prefer not to say)

^b^Category ‘not wanted, not planned’removed due to empty cells

Table 3 shows the distribution of CAM use by pregnant women receiving midwifery care per type of CAM practitioner. Manual therapists were visited most frequently (4.1 %), followed by acupuncturists (1.9 %). CAM practitioners were mostly visited 1–3 times, except for acupuncturists (4–6 times).

**Table 3:** Distribution of CAM use by pregnant women receiving primary midwifery care per type of CAM practitioner (N = 1500)

CAM practitioners	Number of women (%)	Mode of frequency of consultation (if visiting)	Range (if visiting)
Acupuncturist	28 (1.9)	4–6	1–3, >15
Anthroposophical practitioner	6 (0.4)	1–3	1–3, >15
Homeopath	24 (1.6)	1–3	1–3, 10–12
Manual therapist*	62 (4.1)	1–3	1–3, >15
Naturopath	8 (0.5)	1–3	1–3, 7–9
Paranormal practitioner	8 (0.5)	1–3	1–3, >15
Other alternative practitioner**	29 (1.9)	1–3	1–3, >15

*Osteopath, chiropractor, manual therapist

**For example: shiatsu therapy, reflexology, Ayurvedic Medicine, iridology, haptonomy, kinesiology, or Analytical-Synthetical Response therapy

Table 4 shows the associations of predisposing, enabling, need and health behaviour characteristics with use of CAM. Regarding *enabling characteristics*, our analyses showed that women with supplementary health care insurance were three times more likely to visit a CAM practitioner compared to women with only basic health care insurance (adjusted OR = 3.11; 95 % CI 1.41-6.85; see Table 4). With respect to *need variables*, women who rated their health as ‘bad/fair’ were 2.6 times more likely to visit a CAM practitioner compared to women who rated their health as ‘good’. Furthermore, women who reported a chronic illness or handicap were more likely to visit a CAM practitioner than women reporting no chronic illness or handicap (OR = 1.93). Regarding *health behaviour variables*, women who smoked (compared to non-smokers), and women using alcohol during pregnancy (compared to non-drinking women) were more likely to visit a CAM practitioner.

**Table 4:** Associations of predisposing, enabling, need and health behaviour characteristics with use of CAM (N = 1500): odds ratios (OR) and 95 % confidence intervals (CI)

	Crude OR (95 % CI)	Adjusted OR (95 % CI)^a^
Predisposing variables		
Age (years)		
≤20	0.78 (0.10–6.06)	
≥36	1.15 (0.71–1.88)	
21–35	1.00 (ref.)	
Ethnicity		
Non-western	1.01 (0.42–2.40)	
Western non-Dutch	1.43 (0.79–2.61)	
Native Dutch	1.00 (ref.)	
Marital status		
Living alone	1.28 (0.44–3.72)	
Married or living together	1.00 (ref.)	
Occupation		
Unemployed	1.14 (0.70–1.86)	
Disabled	1.46 (0.32–6.60)	
Employed	1.00 (ref.)	
Educational level		
Middle	1.25 (0.63–2.50)	
High	1.62 (0.84–3.12)	
Low	1.00 (ref.)	
Intended place of delivery		
Hospital/birth centre midwifery-led	1.19 (0.83–1.71)	
Hospital consultant-led	1.99 (0.56–7.10)	
Home	1.00 (ref.)	
Religion		
No	1.11 (0.77–1.60)	
Yes	1.00 (ref.)	
Enabling variables		
Basic and supplementary health care insurance		
Basic and supplementary	2.92 (1.34–6.36)	3.11 (1.41–6.85)
Basic	1.00 (ref.)	1.00 (ref.)
Net household income		
> €2000	1.65 (0.86–3.14)	
< €2000	1.00 (ref.)	
Accessibility of care (phone)		
Problems	1.05 (0.66–1.66)	
No problems	1.00 (ref.)	
Accessibility of care (getting to and from the practice)		
Problems	1.04 (0.44–2.48)	
No problems	1.00 (ref.)	
Need variables		
General self-rated health		
Excellent/Very good	1.07 (0.72–1.60)	1.29 (0.91–1.82)
Bad/Fair	2.81 (1.78–4.43)	2.63 (1.65–4.21)
Good	1.00 (ref.)	1.00 (ref.)
Quality of life (EuroQol)		
Poor health status	1.84 (1.26–2.70)	
Good health status	1.00 (ref.)	
Chronic illnesses or handicaps		
Yes	2.36 (1.44–3.87)	1.93 (1.14-3.27)
No	1.00 (ref.)	1.00 (ref.)
PRAQ*-Child		
Fear	1.08 (0.75–1.56)	
No fear	1.00 (ref.)	
PRAQ*-Delivery		
Fear	1.78 (0.51–6.24)	
No fear	1.00 (ref.)	
PRAQ*-Body		
Fear	1.26 (0.86–1.84)	
No fear	1.00 (ref.)	
Planned and wantedness of pregnancy**		
Wanted, not planned	1.18 (0.74–1.89)	
Planned and wanted	1.00 (ref.)	
BMI		
≤18.5	1.67 (0.68–4.09)	
25–< 30	1.02 (0.64–1.61)	
≥30	0.73 (0.29–1.85)	
18.5–< 25	1.00 (ref.)	
Parity		
Primi/multiparous	1.11 (0.78–1.58)	
Nulliparous	1.00 (ref.)	
Difference between number of pregnancies and number of births		
≥2	1.15 (0.77–1.71)	
1	1.00 (ref.)	
Health behaviour variables		
Locus of control		
No	0.77 (0.42–1.40)	
Yes	1.00 (ref.)	
Folic acid use		
No	0.91 (0.44–1.89)	
Yes, inadequately	1.20 (0.83–1.72)	
Yes, adequately	1.00 (ref.)	
Smoking		
Yes	1.88 (1.08–3.27)	1.88 (1.06–3.33)
No	1.00 (ref.)	1.00 (ref.)
Passive smoking		
Yes	1.22 (0.62–2.42)	
No	1.00 (ref.)	
Alcohol use		
Yes	2.28 (1.46–3.56)	2.30 (1.46-3.63)
No	1.00 (ref.)	1.00 (ref)
Health care utilization in Midwifery Practice		
Inadequate	1.16 (0.76–1.76)	
Adequate plus	0.66 (0.28–1.55)	
Adequate	1.00 (ref.)	

^a^ = Corrected for all other variables in the adjusted model, predictors were considered in the final model if p-value was < 0.05

*PRAQ = Pregnancy Related Anxiety Questionnaire

**category ‘not wanted, not planned’ removed due to empty cells

## Discussion

We assessed the prevalence and determinants of CAM practitioner use of low-risk pregnant women in primary midwifery practices in the Netherlands, and found a prevalence of 9.4 % CAM practitioner use. Low-risk pregnant women were more likely to visit a CAM practitioner if they had supplementary health care insurance, if they rated their health as ‘bad/fair’, if they reported a chronic illness or handicap, if they smoked during pregnancy, and if they used alcohol during pregnancy.

### Interpretation

We found a rate of almost 10 % of pregnant women consulting a CAM practitioner, fitting into the range reported in the literature [5, 25]. We expected to find a lower prevalence compared to that in the general female population because of the composition of our research population consisting of low-risk pregnant and as a consequence, healthy women. However, contrary to our expectations, we found a higher prevalence than among the general population of women in the Netherlands (9.4 % vs 7.5 %) [26]. This difference might indicate the relatively large need of pregnant women for additional care besides regular pregnancy care, which might be related to women having concomitant health problems affected by pregnancy (i.e., nausea, back problems).

In accordance with the general female population in the Netherlands, osteopaths and chiropractors were the most consulted CAM practitioners in our study [26]. An explanation may be that musculoskeletal problems are common in pregnancy, varying in severity from mild to a severe [27], leading to an increased use of these manual therapists. In addition, pregnant women may presume that manual therapy potentially provides a safe alternative to pain medication during pregnancy, for example in the case of low back and pelvic pain [28, 29].

Regarding *need variables*, we found that women rating their health as ‘bad/fair’ and women reporting a chronic illness were more likely to visit a CAM practitioner. This also holds for the general population with chronic illnesses in the Netherlands [26]. It is possible that women with chronic illnesses look for comfort measures or symptom management which they cannot find in conventional medicine or midwifery care [28, 30].

Regarding *health behaviour variables*, we found an association between smoking, alcohol use and CAM practitioner use. This finding conflicts with findings from international studies on this topic [31]. However, these studies mostly concerned non-pregnant women. In pregnant women CAM practitioner use may be a coping strategy reflecting the intention to stop drinking and/or smoking [15]. Moreover, these health behaviour adjustments in pregnancy can cause prenatal psychological distress, which also is associated with CAM use [30, 32].

Surprisingly, we did not find any significant associations of predisposing variables with CAM practitioner use. In the literature, a higher educational level has been shown to be associated with CAM use [6, 33]. It is assumed that higher educational level may encourage the development of critical thinking, which may lead to the appraisal of health care options that lie outside conventional care [1]. Our descriptives (Table 2) show that 62.4 % of highly educated women visit a CAM practitioner as compared to 54.4 % of non-users. However, when we controlled for many variables this difference was not statistically significant. We have no indication that the educational level of Dutch women differs substantially from women in other countries. An explanation may be that the variation in educational level among CAM users was not large enough to establish a statistically significant association. Bishop et al.[33] found that older mothers were more likely to consult a CAM practitioner compared to younger mothers. However, the effect size of this association seems to be small. In our research there was a slight difference between CAM users and non-CAM users (15.6 % vs 13.3 %) in the group of women aged 36 years and over. When we corrected for many other variables, age was not significantly related to CAM use.

### Strengths and limitations

One strength of this research is the use of a unique and large sample of women who had low-risk pregnancies. This allowed us to carry out the study in a homogeneous population in primary midwifery care. Furthermore, we used data of a large study population, which covered all components of the behavioural model of Andersen.

Our study population included slightly more highly educated and native-Dutch women compared to the general Dutch population [26]. However, educational level and ethnic background were not significantly associated with the outcome measures. Potentially, recall bias may have occurred due to the timing of the completion of the third questionnaire for the DELIVER database. However, we attempted to reduce this risk by only including women who had filled in this questionnaire up to 13 weeks postpartum. Moreover, it seems likely that most women will remember whether they visited a CAM practitioner or not. Next to this, self-report can create bias due to social desirability. However, pregnant women could fill in the questionnaire on CAM use with confidentiality, which might decrease non-disclosure because of the absence of a potentially judgmental health care provider. Finally, we determined a group consisting of all manual therapists. We chose to do this because of the similarities of these professions in treating patients with spine problems. As a result, we do not have outcomes regarding the specific practitioners in this group of manual therapists.

### Implications

The results of this research indicate that CAM use is relatively high even in a low-risk population of pregnant women. This raises the question of how maternal care providers can become more aware of CAM use by their clients. Midwives and obstetricians must be attentive to CAM use. We know that non-disclosure can occur for different reasons [12]. Therefore, we advise midwives/obstetricians to actively ask their clients whether they have contacted a CAM practitioner at every scheduled consultation. Furthermore, our findings reflect the need for informing and collaborative care approaches by all practitioners involved in the care of the same pregnant woman.

Our research shows that it is necessary for midwives to learn about CAM, which is not commonly included in midwifery education [34]. This may consist of acquiring knowledge about CAM and learning how to identify safety issues regarding maternal health care. In addition, midwives have to encourage pregnant women to make use of professional bodies and voluntary registers if considering using CAM. For instance, in Great Britain this would be the Complementary and Natural Healthcare Council (CNHC).

Research challenges concern, specifically, understanding the reasons, attitudes and beliefs of low-risk women who consult CAM practitioners. Why do pregnant women consult CAM practitioners in addition to regular pregnancy care practitioners? Which CAM practitioners are mostly consulted? Is it used as a supplement to or a substitute for traditional care? Maternal health care practitioners can use this information to better meet the needs of pregnant women [30].

## Conclusions

CAM is relatively frequently used in a sample of low-risk pregnant women. The determinants of this use as revealed in this study diverge from those found in other studies using more heterogeneous populations. Maternal health care practitioners must become more aware of CAM practitioner use and incorporate this knowledge into daily practice, actively discussing this subject with pregnant women.

## Abbreviations

GP: General practitioner
CAM: Complementary and alternative medicine
DELIVER: Data primary care delivery
OR: Odds ratio
CI: Confidence interval

## References

[1] Frawley J, Adams J, Sibbritt D, Steel A, Broom A, Gallois C (2013). Prevalence and determinants of complementary and alternative medicine use during pregnancy: Results from a nationally representative sample of Australian pregnant women. Aust N Z J Obstet Gynaecol.

[2] Strouss L, Mackley A, Guillen U, Paul DA, Locke R (2014). Complementary and Alternative Medicine use in women during pregnancy: do their healthcare providers know?. BMC Complement Altern Med.

[3] Mitchell M (2010). Risk, pregnancy and complementary and alternative medicine. Complement Ther Clin Pract.

[4] Hall HG, Griffiths DL, McKenna LG (2011). The use of complementary and alternative medicine by pregnant women: A literature review. Midwifery.

[5] Adams J, Lui CW, Sibbritt D, Broom A, Wardle J, Homer C (2009). Women’s use of complementary and alternative medicine during pregnancy: a critical review of the literature. Birth.

[6] Steel A, Adams J, Sibbritt D, Broom A, Gallois C, Frawley J (2014). Determinants of women consulting with a complementary and alternative medicine practitioner for pregnancy-related health conditions. Women Health.

[7] Thomson P, Jones J, Browne M, Leslie SJ. Why people seek complementary and alternative medicine before conventional medical treatment: A population based study. Complement Ther Clin Pract. 2014, in press(0). 10.1016/j.ctcp.2014.07.008 25156988

[8] Brocklehurst P, Hardy P, Hollowell J, Linsell L, Macfarlane A, Birthplace in England Collaborative Group (2011). Perinatal and maternal outcomes by planned place of birth for healthy women with low risk pregnancies: the Birthplace in England national prospective cohort study. BMJ.

[9] PRN foundation (2013). Netherlands Perinatal Registry.

[10] De Geus M (2012). Midwifery in the Netherlands.

[11] D’Crus A, Wilkinson JM (2005). Reasons for choosing and complying with complementary health care: an in-house study on a South Australian clinic. J Altern Complement Med.

[12] Thomson P, Jones J, Evans JM, Leslie SL (2012). Factors influencing the use of complementary and alternative medicine and whether patients inform their primary care physician. Complement Ther Med.

[13] Holst L, Wright D, Haavik S, Nordeng H (2011). Safety and efficacy of herbal remedies in obstetrics—review and clinical implications. Midwifery.

[14] Lim A, Cranswick N, South M (2011). Adverse events associated with the use of complementary and alternative medicine in children. Arch Dis Child.

[15] Steel A, Adams J, Sibbritt D, Broom A, Frawley J, Gallois C. Relationship between complementary and alternative medicine use and incidence of adverse birth outcomes: An examination of a nationally representative sample of 1835 Australian women. Midwifery. 2014;30:1157-1165. 10.1016/j.midw.2014.03.015 24742636

[16] Viljoen E, Visser J, Koen N, Musekiwa A (2014). A systematic review and meta-analysis of the effect and safety of ginger in the treatment of pregnancy-associated nausea and vomiting. Nutr J.

[17] Andersen RM, Rice TH, Kominski GF (2007). Changing the U.S. health care system; key issues in health services policy and management.

[18] Mannien J, Klomp T, Wiegers T, Pereboom M, Brug J, de Jonge A (2012). Evaluation of primary care midwifery in the Netherlands: design and rationale of a dynamic cohort study (DELIVER). BMC Health Serv Res.

[19] Herdman M, Gudex C, Lloyd A, Janssen M, Kind P, Parkin D (2011). Development and preliminary testing of the new five-level version of EQ-5D (EQ-5D-5 L). Qual Life Res.

[20] Huizink AC, Mulder EJ, de Medina PG R, Visser GH, Buitelaar JK (2004). Is pregnancy anxiety a distinctive syndrome?. Early Hum Dev.

[21] World Health Organization. [http://www.who.int/mediacentre/factsheets/fs311/en/].

[22] Health Council of the Netherlands (2008). Towards an optimal use of folic acid.

[23] Kotelchuck M (1994). The Adequacy of Prenatal Care Utilization Index: Its US distribution and association with low birthweight. Am J Public Health.

[24] Goldstein H, Browne W, Rasbash J (2002). Multilevel modelling of medical data. Stat Med.

[25] Hall HG, McKenna LG, Griffiths DL (2012). Midwives’ support for Complementary and Alternative Medicine: A literature review. Women Birth.

[26] Statistics Netherlands. [http://www.cbs.nl/nl-NL/menu/themas/gezondheid-welzijn/publicaties/artikelen/archief/2014/2014-4041-wm.htm].

[27] Keriakos R, Bhatta SRC, Morris F, Mason S, Buckley S (2011). Pelvic girdle pain during pregnancy and puerperium. J Obstet Gynaecol.

[28] Close C, Sinclair M, Liddle SD, Madden E, McCullough JE, Hughes C. A systematic review investigating the effectiveness of Complementary and Alternative Medicine (CAM) for the management of low back and/or pelvic pain (LBPP) in pregnancy. J Adv Nurs. 2014;70(8):1702-16. 10.1111/jan.12360 24605910

[29] Wang SM, DeZinno P, Fermo L, William K, Caldwell-Andrews AA, Bravemen F (2005). Complementary and alternative medicine for low-back pain in pregnancy: a cross-sectional survey. J Altern Complement Med.

[30] Thorne S, Paterson B, Russell C, Schultz A (2002). Complementary/alternative medicine in chronic illness as informed self-care decision making. Int J Nurs Stud.

[31] Al-Windi A (2004). Determinants of complementary alternative medicine (CAM) use. Complement Ther Med.

[32] Furber CM, Garrod D, Maloney E, Lovell K, McGowan L (2009). A qualitative study of mild to moderate psychological distress during pregnancy. Int J Nurs Stud.

[33] Bishop JL, Northstone K, Green JR, Thompson EA (2011). The use of Complementary and Alternative Medicine in pregnancy: data from the Avon Longitudinal Study of Parents and Children (ALSPAC). Complement Ther Med.

[34] Steel A, Adams J (2012). Developing midwifery and complementary medicine collaboration: The potential of interprofessional education?. Complement Ther Clin Pract.

